# Synergic fabrication of multifunctional liposomes nanocomposites for improved radiofrequency ablation combination for liver metastasis cancer therapy

**DOI:** 10.1080/10717544.2021.2008056

**Published:** 2022-02-11

**Authors:** Ning Zhang, Yibin Wu, Weiqi Xu, Zhenjian Li, Lu Wang

**Affiliations:** a Department of Hepatic Surgery, Fudan University Shanghai Cancer Center, Shanghai, China; b 3D Biomedicine Science & Technology Co., Limited, Shanghai, China

**Keywords:** Liposome, doxorubicin, liver cancer, apoptosis, *in vivo* tumor model

## Abstract

The field of biomedical research has recently been interested in nanoplatforms with various functionalities, such as cancer drug carriers and MRI and optical imaging, as well as thermal treatment, among other things. As a result of the present investigation, a unique multifunctional liposome (MFL) was established in this investigation. Using radiofrequency-induced imaging and drug release based on magnetic field impact, a dual drug delivery targeted with tumor multi-mechanism treatment was made more effective. The C60 (fullerene) surface was coated with iron nanocomposites to establish the proposed nanosystems, and PEGylation was used (Fe_3_O_4_-C60-PEG_2000_). For fullerene radiofrequency-triggered drug release, thermosensitive DPPC liposomes with folate-DSPE-PEG_2000_ enveloped the binary nanosystems and doxorubicin (DOX). The *in vitro* cytotoxicity of the nanocomposites was confirmed by the liver metastasis in HT-29 colon cancer cells using radiofrequency. The flow cytometry analysis confirmed the apoptosis cell death mechanism. The thermal treatment combined chemotherapeutic MFL nano framework transformed radiofrequency radiation from thermoresponsive liposomes, which was noticed both *in vivo* and *in vitro*. Due to their superior active tumor targeting and magnetic targeting characteristics, the MFL could also selectively destroy cancerous liver cells in highly co-localized targets.

## 1. Introduction

As a deadly disease with a high prevalence, liver cancer demands special attention. We are surprised by traditional chemotherapy, which has a significant level of tissue cytotoxicity (Ferreira et al., 2020; Khalifehzadeh & Arami, 2020; Yang et al., 2021). The use of polymeric nanoparticles, liposomes, and inorganic material nanocomposites is now being investigated as new nano-drug delivery methods for liver cancer. Due to the increased permeability and retention impact, these forms can enhance oral absorption in the plasma and passively target tumor tissues (Farzin et al., 2020). Cancer cells do not get enough chemotherapeutic drugs despite passive targeting, which lowers adverse effects. Different liver-targeting molecules have been implanted on the carriers' surfaces to overcome low target efficiency (Fan et al., 2020). The ligands are folic acid (FA), galactose, protein, hyaluronic acid (HA), and glycyrrhetinic acid. The anticancer impact can be maximized by entering tumor tissues via receptor-mediated endocytosis using ligand-modified nano-drug delivery devices (Marques et al., 2020).

They are one of the most sophisticated methods of delivering cytotoxic drugs. Oncologists have authorized the clinical usage of a liposome-based DOX formulation that outperformed free DOX on the therapeutic index (Ledezma-Gallegos et al., 2020; Patel et al., 2020; Barani et al., 2021). Targeting receptors and stimuli-sensitive release inside the tumor can improve the therapeutic effectiveness of liposomal compositions. Hence, it is no wonder that tailored and temperature-sensitive formulas are so popular. Targeting folate receptors in liposomal delivery of drugs to cancerous cells is promising and successful *in vitro* (Vu et al., 2020). A non-targeted stealth liposome formulation, on the other hand, did not affect the drug concentrations in tumors. The FA receptors are overexpressed in many cancerous cells, comprising those in the kidney, liver, brain, ovary, colon, prostate, and lung. More FA can transfect HT-29 metastasis liver cancer cells, which are positive folate receptors (Chowdhury et al., 2020; Gu et al., 2020; Sonju et al., 2021). HT-29 metastasis liver cancer cells and HT-29 cells do exhibit significant levels of the folate receptor. It is well-known that magnetic nanocomposites have enormous promise in the domains of medication delivery, cancer diagnosis and therapy, and cancer diagnostics and treatment (Ding et al., 2020; Kim et al., 2020; Naumenko et al., 2021). According to a recent study, iron oxide magnetite nanocomposites (MNPs) have been broadly used in pharmaceutical fields because of their high biocompatibility and low toxicity. MNPs with ultrathin particle sizes and extreme magnetizations levels may be controlled by a magnetization to penetrate human tissues, suggesting use for magnetic therapy. The inherent magnetic characteristics of MNPs for drug administration have garnered substantial attention among the vast spectrum of nanomaterials being explored for biomedical applications (Lv et al., 2020). A combination of radiofrequency thermal and drug delivery treatment for cancer may be possible with thermosensitive liposomes. When a solid permanent gradient magnetic field is applied to a target tissue, such as a tumor, MNPs are utilized as potential drug delivery carriers that concentrate in that tissue. T_2_ (spin–spin relaxation) imaging can also be employed with magnetic liposomes to track their biodistribution *in vivo* noninvasively using MRI. These nanocomposites are inoculated into the cancer cells and subjected to rotating magnetic fields for this purpose (Dana et al., 2020; Mansoori et al., 2020; Swami Vetha et al., 2020).

Thermal ablation of cancerous cells is achieved by using radiofrequency thermal treatment (RTT), which converts radiofrequency irradiation energy into heat using RF absorbing agents (Jose et al., 2018; Löffler et al., 2019; Rangamuwa et al., 2021). As a noninvasive, controlled, and highly effective therapeutic technique, RTT has attracted a lot of interest in recent years. Radiation from the RF activation system may enter tissues with low energy loss, making it an attractive therapeutic option (Chung et al., 2017; Mauri et al., 2019; Paulides et al., 2020). C60 possesses unusual electrical and chemical characteristics, which led us to anticipate in this work that functionalized C60 would produce substantial amounts of heat when exposed to concentrated external RF fields in the region of 13.56 MHz, which allow them to serve effectively as an antitumor therapeutic. Fullerene's heat produced by a 13.56 MHz radio frequency would induce the release of chemotherapeutic drugs (Oberacker et al., 2020). The formulations must erupt from the systemic blood-circulation into the tumor tissues to achieve receptor-mediated endocytosis (Bale et al., 2019). Accordingly, we use liposomal formulations with shielded liposomes that have been shown to circulate for an extended period and to erupt due to the enhanced permeability retention (EPR) effect. Finally, adequate to guarantee that the formulation remains in the tumor for as long as possible (Shoji et al., 2017). Magnetic field aiming would help and speed up these stages by aiding and accelerating processes. The probability increases the tumor cell absorption of receptor-mediated drugs by gradient magnetic field targeting and the ultimate phase of cancer cell death through the radiofrequency ablation of intracellular drug release (Palussière et al., 2018; Han et al., 2020; Wust et al., 2020).

**Figure 1.**
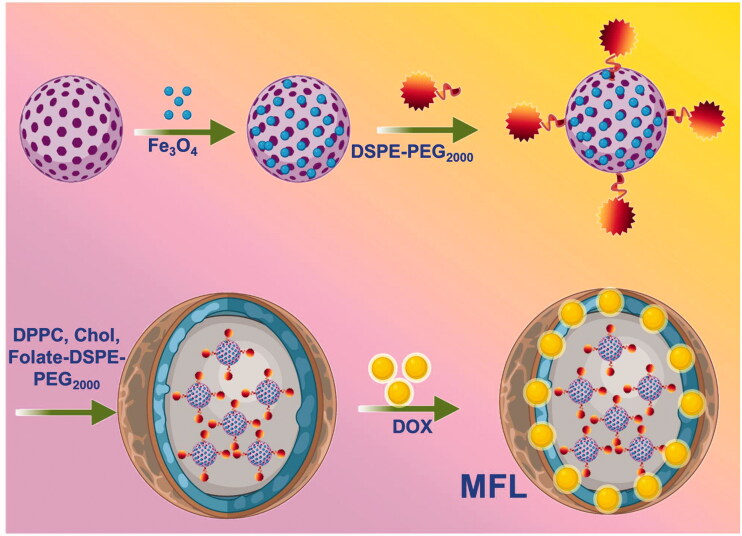
Schematic representation and fabrication of multifunctional liposome (MFL) drug release by radiofrequency ablation (RF) process.

**Figure 2.**
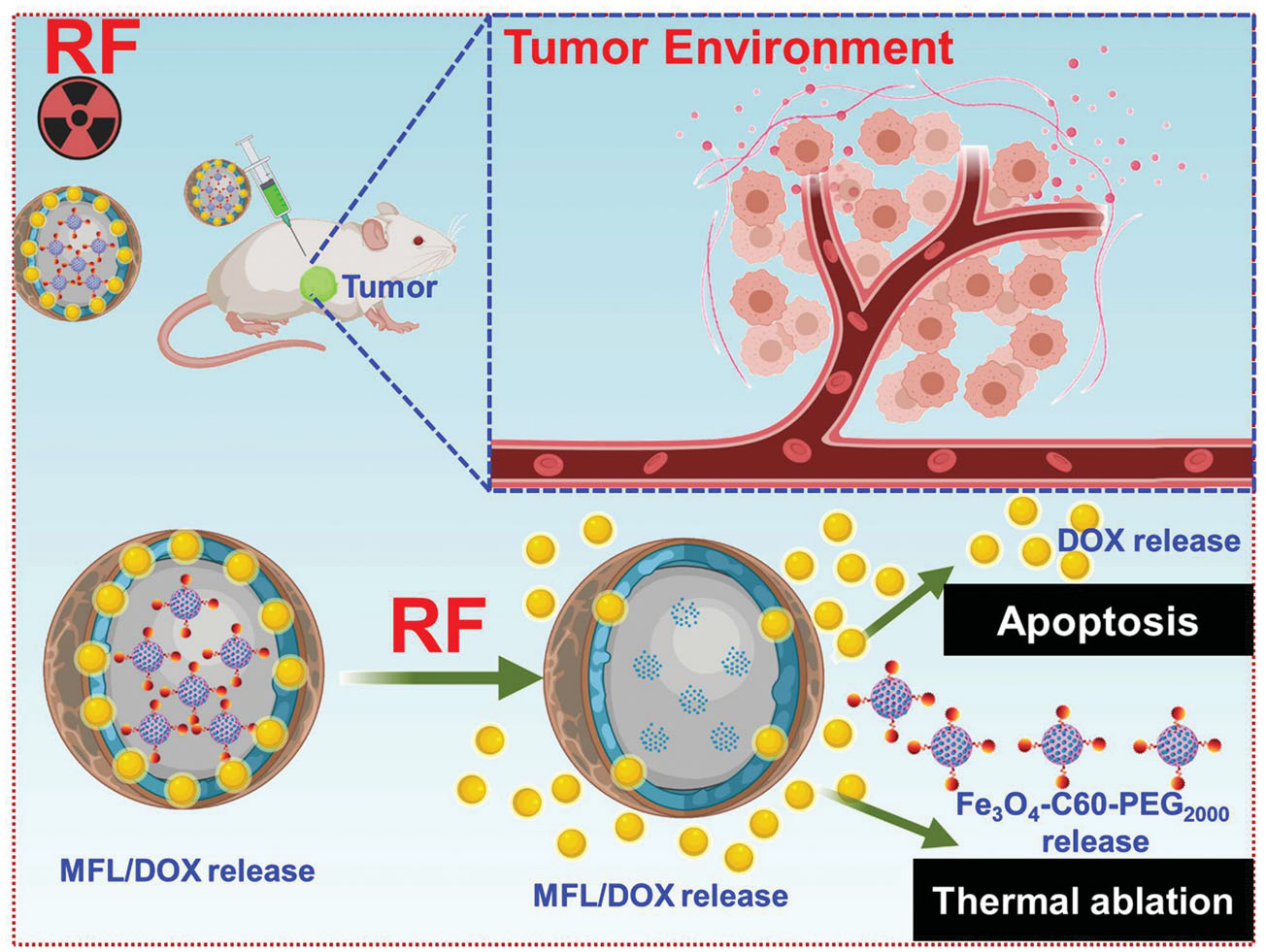
Radiofrequency ablation (RF) of chemotherapy and radiotherapy method.

Metastases result from a multi-step process in which cancerous cells separate from the primary tumor and then spread to new sites throughout the body. Most cancer-related deaths are caused by metastasis, as has been widely reported. The treatment approach is determined by the presence or absence of metastatic masses and the necessity to prevent the spread of malignant cells. In cancer diagnosis and therapy, the approach of using distinct individual functions is widespread and established. We developed thermoresponsive liposomes incorporating fullerene and magnetic iron oxide nanoparticles to facilitate the treatment by integrating several functionalities into a single nanosystem. We aim to establish a multifunctional nanoplatform for sustained delivery, dual drug delivery targeted. Together, these advantages have the potential to act as cancer nanomedicine agents. These hybrid nanosystems were coated with folate receptors targeting thermosensitive liposomes and DOX after being produced with C60-Fe_3_O_4_ and functionalized with polyethylene glycol (PEG_2000_). There are 80:20:5:4 ratios in the optimized liposome formulations, consisting of DPPC/DSPC/DSPE-PEG_2000_-folate@DOX. High resolution-transmission electron microscopy (HR-TEM) and dynamic light scattering were used to evaluate a tumor-focused liposome with multifunctional features for RTT and controlled release profile (Figures 1 and 2). Cancer cells and tumor-bearing *in vivo* animal models were used to examine the nanosystems' RTT and tumor-specific effectiveness. The multifunctional liposome (MFL) nanosystems fabricated in this study have promise for cancer nanomedicine applications.

## 2. Experimental

### 2.1. Materials

All the lipids were provided by Avanti Polar Lipids (Alabaster, AL). Doxorubicin (DOX, purify ≥98.0%) and FA (purify ≥96.0%) were purchased from Bio Basic Inc. (Markham, Canada). N,N′-dicyclohexylcarbodiimide (DCC), N-hydroxysuccinimide (NHS), and N,N′-disuccinimidyl carbonate (DSC) were purchased from Sigma-Aldrich Co., Ltd. (St. Louis, MO). Dichlorofluorescein diacetate (DCFH-DA) was obtained from Molecular Probes Inc. (Eugene, OR). 3-(4,5-Dimethylthiazol-2-yl)-2,5-diphenyltetrazolium bromide (MTT) was purchased from Amresco (Solon, OH). Dulbecco's modified Eagle's medium (DMEM) and 0.25% trypsin were purchased from M&C Gene Technology (Beijing, China). Penicillin–streptomycin and trypsin–ethylenediamine tetra-acetic acid (EDTA) were Hyclone (Logan, UT). Fetal bovine serum (FBS) was purchased from Gibco Life Technologies (AG, Basel, Switzerland).

### 2.2. Fabrication of Fe_3_O_4_-C60-PEG_2000_

Fifty milliliters anhydrous toluene solution with NaH and 50 mg of C60 was mixed with 0.2 mL of diethyl bromomalonate in toluene, added drop-by-drop. To get the malonate derivatives of C60, the mixture was stirred at room temperature for 5 h under N_2_. Anhydrous toluene (30 mL), 50 mg of malonate derivative of C60 and 180 mg of NaH, and 20 mL of concentrated HCl were added to the reaction precipitate before the toluene was removed. A conc. HCl solution was used to dissolve the residue, purified, and rinsed with water and methanol. The resultant residues were dissolved in methanol, and the unsolvable residues were separated by filtering off the insoluble components. Solids were air-dried in a vacuum pump at 45 °C for 24 h after they had evaporated.

A combination of 0.5 mL of ethylene glycol (EG) and diethylene glycol was used to dissolve 50 mg of C60-COOH, 270 mg of FeCl_3_·6H_2_O, and 750 mg of sodium acetate (NaOAc). The C60-Fe_3_O_4_ was washed multiple times using ethanol and deionized water before being vacuum dried for 12 h. It was then moved to an autoclave lined with Teflon and heated at 200 °C for 10 h.

As a final stage, we added 60 mg of NH_2_-PEG_2000_-NH_2_ with 15 mL Fe_3_O_4_-C60 solution and ultrasonicated for 10 min. There was added 6 mg of EDC·HCl to the exact solutions. The reaction mixture was magnetically stirred at RT for 24 h before being used. For purification, the resultant product was washed thrice with DD-water, passed via a membrane filter to eliminate unreacted PEG_2000_, and air-dried in a vacuum pump at 45 °C for 12 h after they had evaporated.

### 2.3. DOX adsorption on Fe_3_O_4_-C60-PEG_2000_

For 2 h, 50 mg of Fe_3_O_4_-C60-PEG_2000_ was added to 50 mL of the ethanol–water (1:1) that included 150 mg of DOX and used an ultrasonic cell disruption device to disperse my product after evaporation to remove the water and ethanol, and then dispersed it in 20 mL of water (10 times, 400 W). For DOX removal, the nanosuspensions were centrifuged. It was necessary to keep the Fe_3_O_4_-C60-PEG_2000_@DOX nanosuspension until usage at 4 °C.

### 2.4. Synthesis of DOX-loaded liposomes

After the liposomes were synthesized using the thin-film dispersion technique (molar ratio 80:20:5:4), they were sized using sonography and membrane extrusion (Wang et al., 2011; Unnam et al., 2019; Xiao et al., 2021). In a round bottom flask, 25 mg of total lipids (folate-DPPC/DSPC/DSPE-PEG_2000_@DOX) was mixed with chloroform–diethyl ether mixture (2:1) and evaporated at 60 °C using a rotary evaporator. The resulting liposomes contained 5 mg of DOX. A rotary evaporator was used to evaporate the solvent, resulting in a thin lipid coating in the flask's circular bottom. It was then dissolved in 1 mL of phosphate buffer with 1 mg of Fe_3_O_4_-C60-PEG_2000_ powder (100 mM, pH 7.4). At 60 °C for 20 min, a rotary evaporator spinning at 120 rpm at atmospheric pressure dried the thin lipid layer in this suspension. Utilizing an extruder (Whatman Inc., Piscataway, NJ) to modify the liposome size, the resultant fabrication was sonicated for 10 min. It was then centrifuged for 30 min at 12,000×*g* in a millipore to remove DOX that had not been encapsulated. Triton X-100 was added to the filtered liposomes to cause the membrane to break. The result was then diluted with an anhydrous ethanolic solution and further sonicated to verify that DOX was fully dispersed, then centrifuged to distinct Fe_3_O_4_-C60-PEG_2000_@DOX.

### 2.5. Characterizations of liposomes

Fourier-transform infrared (FT-IR) spectra of lyophilized samples were recorded using a Nicolet 5700 FT-IR spectrometer (Thermo Electron Corporation, Waltham, MA). The morphology images of nanocomposites were recorded by transmission electron microscopy (TEM) on a Hitachi 7700A electron microscope (120 kV). The average particle sizes and *ζ*-potentials of nanocomposites were evaluated by a size analyzer (Zetasizer ZS90, Malvern, UK). The absorption spectra were measured using a microplate reader (Multiskan GO).

### 2.6. DOX release profile from liposomes

A dialysis technique was used to examine DOX release *in vitro*. Dialysis bags (MW 8000 Da) were filled with samples of DOX, Fe3O4-C60-PEG2000@DOX, and MFLs, which were then added to 50 mL release medium. A 150 rpm min1 stirring rate was used in the release experiment, which was carried out at 37 °C and 42 °C. 0.5% of SDS was added to PBS (pH 7.4) as the release medium. Dialysis bags were emptied and replaced with new release media at intervals of 0.2 mL. It was determined that nanosystems discharged a significant amount of DOX into the medium utilizing high-performance liquid chromatographic (HPLC) under the chromatographic environments (Zhang et al., 2016, 2017; Ding et al., 2020).

### 2.7. Culture of the liver cancer cells

Liver metastasis in HT-29 colon cancer was cultured in a DMEM medium. All the cell lines were obtained from American Type Culture Collection (ATCC) and grew at the culture media supplemented with 10% FBS and 1% antibiotics (100 U/mL penicillin and 100 U/mL streptomycin). The cells were cultured in an incubator (Thermo Scientific, Waltham, MA) at 37 °C under a humidified atmosphere containing 5% CO_2_.

### 2.8. *In vitro* RTT treatment

A sodium version of sulforhodamine B was used to assess RTT therapy (SRB). Water-soluble SRB is an anionic dye that may be coupled with essential amino acids of composite proteins in acidic circumstances. Its absorption value at 540 nm wavelength is directly proportional to cell uptake. A density of 3000 cells was plated in a 96-well plate with flat bottoms and incubated for 24 h before being removed. Next, 200 L of new media was added to each well of the plates, along with Fe_3_O_4_-C60-PEG_2000_@DOX and the MFL, and the plates were then subjected to 13.56 MHz (300 W) radiofrequency ablation for 20 min to examine the sensitivity of the RF. Cell viability was determined using the SRB analysis after incubated with 24 h, 48 h, and 72 h in 37 °C for a CO_2_ atmosphere. The MFL underwent the same process without irradiation (Pantano et al., 2017; Chung et al., 2018; Prasad et al., 2018; Beyk & Tavakoli, 2019).

### 2.9. Cellular uptake

Five milliliters of Fe_3_O_4_-C60-PEG_2000_ nanoparticles were subsequently sonicated and shielded from light. A Sephadex G-25 column was used to remove excess FITC (Sigma-Aldrich Co. LLC, St. Louis, MO). A total density of 2 × 10^5^ HT-29 metastasis cells was plated per well on glass coverslips in six-well plates. For 0.5 h, 1 h, 2 h, and 4 h, cells were incubated with Fe_3_O_4_-C60-PEG_2000_@DOX and MFL. On certain days, the cells were rinsed three times with PBS and then 15 min in 4% formaldehyde before being washed with PBS at the stated time points. The cellular uptake fluorescence images were recorded with a CLSM (LSM880, Zeiss, Dresden, Germany).

### 2.10. Examination of intracellular ROS

The dichlorofluorescein diacetate (DCFH-DA) ROS assay kit detected reactive oxygen species (ROS) generation within cells. A density of 5 × 10^4^ of HT-29 metastasis cells per plate was planted in confocal dishes. DCFH-DA was poured into the cells after incubated with Fe_3_O_4_-C60-PEG_2000_@DOX and MFL for various hours (6, 8, 12, and 24). Incubation for 30 minutes was followed by two PBS-washes, followed by 20 minutes of radiofrequency irradiation at 13.56 MHz (300 W). Irradiated cells were imaged with a fluorescent microscope after radiofrequency irradiation at 13.56 MHz. The ROS fluorescence images were recorded with a CLSM (LSM880, Zeiss, Dresden, Germany).

### 2.11. Examination of apoptosis

Evaluation of cell apoptosis utilizes Annexin-V FITC staining kit (Mohamed Subarkhan et al., 2016; Subarkhan & Ramesh, 2016; Mohamed Kasim et al., 2018; Mohamed Subarkhan et al., 2019; Balaji et al., 2020; Sathiya Kamatchi et al., 2020). In the presence of DOX, MFL, Fe_3_O_4_-C60-PEG_2000_@DOX, and MFL/13.56 MHz RF, HT-29 metastasis cell line was incubated at 37 °C for 24 h. Free DOX was utilized as a control in this experiment. It was collected, washed thrice with cold PBS, and subsequently 500 µL binding buffer was added. Five microliters of Annexin V-FITC and five microliters of 3 µL of PI were added and incubated with the cells for 15 min in the dark after increasing the cell density to 1 × 10^6^ cell each well. Flow cytometry analysis was conducted in CytoFLEX (Beckman, Indianapolis, IN).

### 2.12. Xenograft tumor animal model

BALB/C nude mice (4–6 weeks, 16–18 g) were provided by Beijing Vital River Laboratory Animal Technology Co., Ltd. (Beijing, China). Institutional Animal Care and Use Committee (IACUC) of the Department of Hepatic Surgery, Fudan University Shanghai Cancer Center, Shanghai, China, permitted all animal experiments in this investigation. Mice underwent intravenous administration every two days for three times. A total density of 1 × 10^6^ HT-29 metastasis cells injected with mice produced the liver metastasis in HT-29 colon cancer tumor models. As soon as the tumor volume reached 60–100 mm^3^, the mice were utilized. The mice were separated into seven groups (each group seven animals), the disparities in weight and tumor size in each group were reduced as much as possible. Group I: saline group (0.2 mL), group II: free DOX, group III: MFL, group IV: MFL/13.56 MHz RF (20 min, 300 W), group V: MFL/magnet, group VI: Fe_3_O_4_-C60-PEG_2000_@DOX/magnet/13.56 MHz RF (20 min, 300 W), and group VII: MFL/magnet/13.56 MHz RF (20 min, 300 W). A caliper was used every other day to check for clinical signs in the mice, and the tumor size was estimated as volume=(length of tumor)/(width of tumor)_2/2_. As soon as the mice had been treated for 14 days, they were killed, and their organs were removed. The organs were preserved in a 4% formaldehyde solution, fixed in 10% paraffin, and then split into two sections for H&E staining. The H&E images were recorded with a CLSM (LSM880, Zeiss, Dresden, Germany).

### 2.13. Pharmacokinetic examination

After treatment with Fe_3_O_4_-C60-PEG_2000_@DOX and MFL or DOX (DOX dose: 5 mg/kg) for 0.083 h, 0.25 h, 0.5 h, 1 h, 2 h, 4 h, 8 h, and 12 h, blood (0.5 mL) was collected from the mice eyes of free tumor and healthy mice and centrifuged (Sarangi et al., 2020; Yang et al., 2020; Mignani et al., 2021). There were five 5 mL centrifuge tubes filled with the supernatant (0.2 mL). After vortexing, 2 mL of methylated-butyl ether was added to the glass tube and centrifugated. To dissolve DOX in the supernatant, 0.1 mL of ethanol was added, and HPLC determined the presence in blood samples (DOX).

### 2.14. Biodistribution assessment

After 12 days of observation, mice with cancerous tumors were given water ad libitum before treatment. There was a DOX dose of 5 mg/kg in the control group and a Fe_3_O_4_-C60-PEG_2000_@DOX/magnet dose in the experimental groups. When a mouse was placed in the magnetic group, it had a magnet put over its tumor. After 0.5 h, 1 h, 3 h, 6 h, and 12 h of treatment, organs were stored, assessed, and homogenized in buffer (1:3). The amount of DOX in the organs was revealed utilizing the previously explained method, and the results were promising. HPLC was used to determine the amount of DOX in tissues under the chromatographic conditions described above (Gulzar et al., 2015; Song et al., 2015; Zhou et al., 2015).

### 2.15. Statistical analysis

At least three independent experiments were performed to obtain the mean ± standard deviation (SD) result. Statistical significance of the data was analyzed by one-way analysis of variance (ANOVA) with Bonferroni’s post-test. The significance level was set at probabilities of ***p< .001, **p< .01, and *p< .05.

## 3. Results and discussion

### 3.1. Fabrication and characterizations of Fe_3_O_4_-C60-PEG_2000_ and multifunctional liposomes

Carboxylic acid (–COOH) was added to the C60 surface to overcome this barrier. Minor changes were made to the Bingel cycloaddition and ester hydrolysis reactions to produce –COOH-C60. Whereas –COOH-C60 remained steady in water for several weeks without accumulation, suggesting that the COOH was successfully introduced onto C60. The surface of C60-Fe_3_O_4_ is not water-soluble and hence unsuitable for biological applications since it contains Fe_3_O_4_. A PEGylation was conducted on C60-Fe_3_O_4_ to enhance its solubility and biocompatibility. The NH_2_ and carboxyl C60-Fe_3_O_4_ group of PEG_2000_-NH_2_ were condensed. It was found that the Fe_3_O_4_-C60-PEG_2000_ mixture was highly stable in water.

FT-IR spectra showed significant –CH (∼1143 cm^−1^), –NH_2_ group I (∼1661 cm^−1^), and NH_2_ group II (∼1610 cm^−1^) peaks of stretching vibrations, indicating that the PEGylation was effective (Figure S1A). C═O (∼1714 cm^−1^) and –OH (∼3420 cm^−1^) were also detected in the spectra of –COOH-C60 (Figure S1A) related to C60 (Figure S1A), indicating that the functionalization's of C60 with –COOH was a successful process. In a hydrothermal method, iron oxide nanocomposites were chemically deposited onto C60 to produce the C60-Fe_3_O_4_ (Figure S1A). Hence, the C60-Fe_3_O_4_ FT-IR spectrum revealed a notable reduction in O–H and C═O peaks, and a new-found peak was formed at Fe–O (∼575 cm^−1^) though this reaction part of the carboxyl in –COOH-C60 was decreased during this reaction (Figure S1A).

TGA was used to determine the relative quantity of PEG grafted onto the surface of C60-Fe_3_O_4_. When heated to 500 °C, PEG deteriorated entirely, and Fe_3_O_4_-C60 and Fe_3_O_4_-C60-PEG_2000_ lost around 6% and 38% of their mass, respectively. The relative quantity of PEG grafted onto C60-Fe_3_O_4_ was, therefore, 32%. Traditional liposomes revealed a phase transition peak at 164.5 °C, while MFL demonstrated a lower transition temperature, with a significant peak at 41.5 °C (Figure S1B and S1C). Liposomes with multiple functionalities have a lower transition temperature because they contain DPPC lipids in the bilayer, resulting in a less well-ordered gel phase in the molecular arrangement.

**Figure 3.**
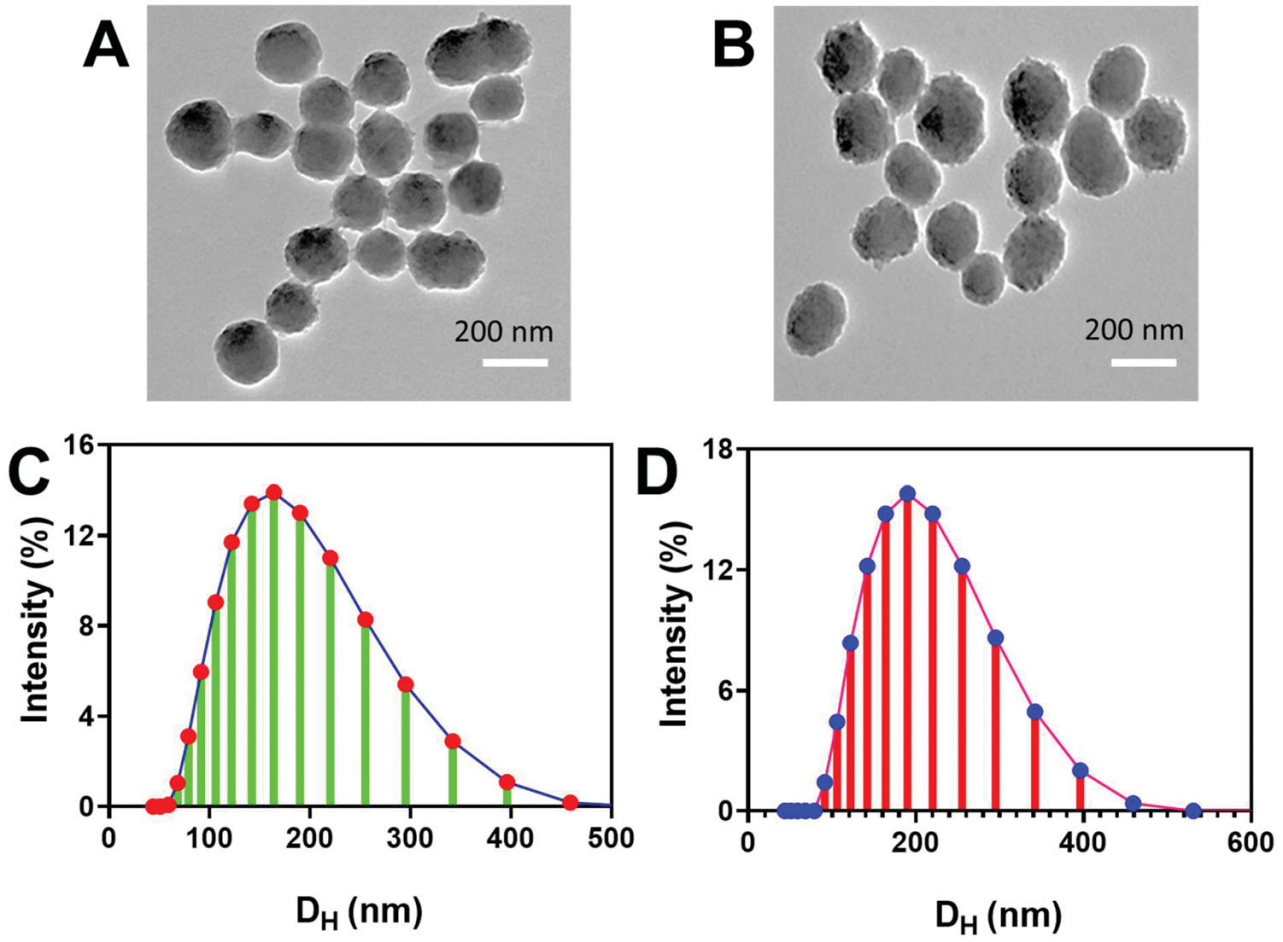
Nanocomposite's morphological examination. (A, B) TEM images of Fe_3_O_4_-C60-PEG_2000_ and MFL. (C, D) Size of the Fe_3_O_4_-C60-PEG_2000_ and MFL.

It was discovered that Fe_3_O_4_-C60-PEG_2000_ aggregates tend to be homodisperse, as verified by DLS and TEM. Fe_3_O_4_-C60-PEG_2000_@DOX measured 170.21 ± 4.3 nm and −34.7 ± 3.1 mV, respectively. These liposomes had a size of 187.0 ± 4.3 nm (Figure 3(A,B)) and electrochemical potential of 333.6 ± 2.1 mV (Figure 3(C)), respectively. TEM was used to determine the morphology of Fe_3_O_4_-C60-PEG_2000_@DOX. Figure 3(B) shows TEM images of Fe_3_O_4_-C60-PEG_2000_@DOX, which indicates that Fe_3_O_4_ was effectively deposited on C60-COOH, as can be observed by the ball-like shape (Figure 3(B)). MFL had a homogeneous size and a well-organized shape, according to TEM images (Figure 3(A,B)). Owing to the extended time necessary for ultrasonic dispersions, the MFL was somewhat larger than Fe_3_O_4_-C60-PEG_2000_.

**Figure 4.**
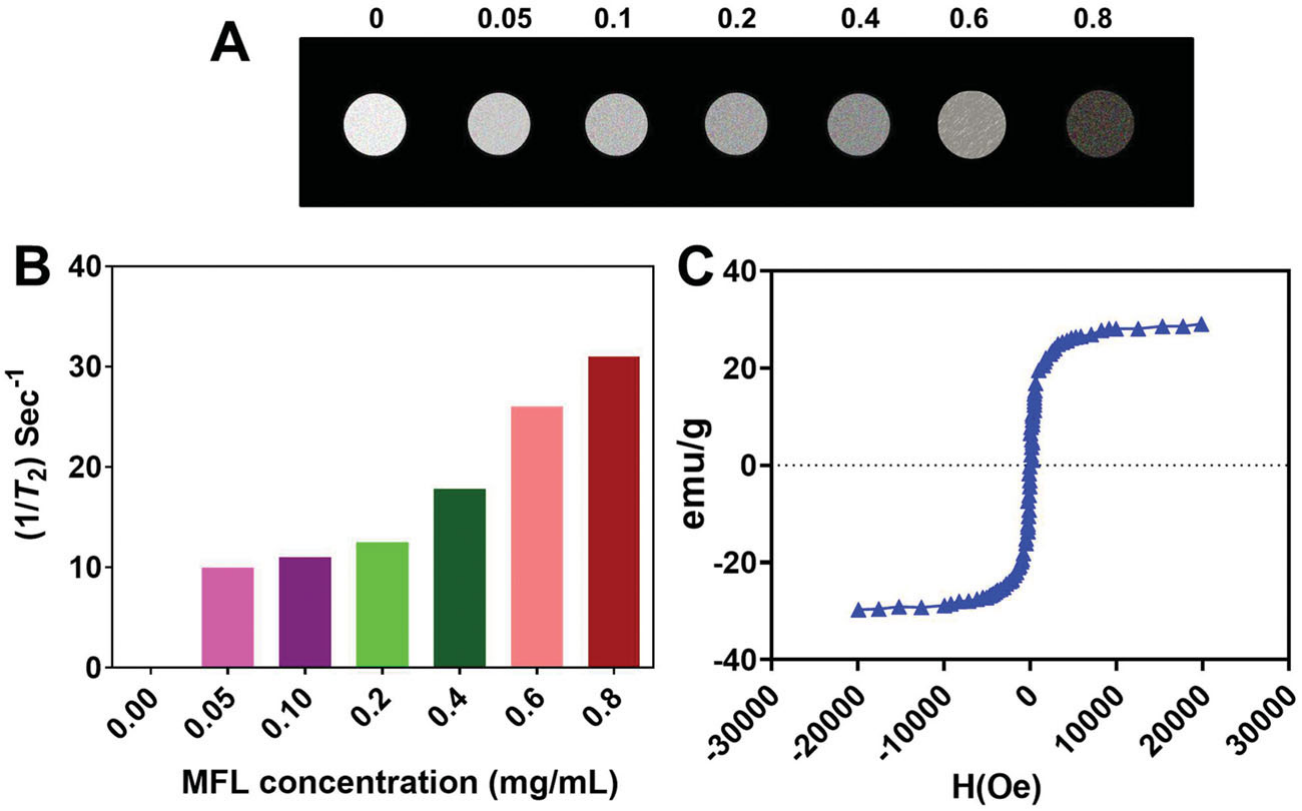
MFL magnetic properties. (A) MR images of the T_2_-weighted MFL at various concentrations. (B) T_2_ relaxation ratio (*R*_2_) of the MFL at various concentrations. (C) Loops of the magnetization.

The magnetic characteristics of the MFL were excellent. For MR imaging, the nanocomposite can be used as a T_2_ contrast agent. T_2_-weighted MRI of the MFL obtained on a 3-T_2_ MR scanning indicated the darkening of concentration-responsive manner. Smaller concentrations of nanocomposites result in brighter T_2_-weighted images, as can be observed (Figure 4(A)). There is 117.29 mg/mL/s1 transverse relaxivity (*R*_2_) in the multi-functional liposome (Figure 4(B)). In addition, the magnetization hysteresis loop revealed that the MFL was superparamagnetic (Figure 4(C)).

**Figure 5.**
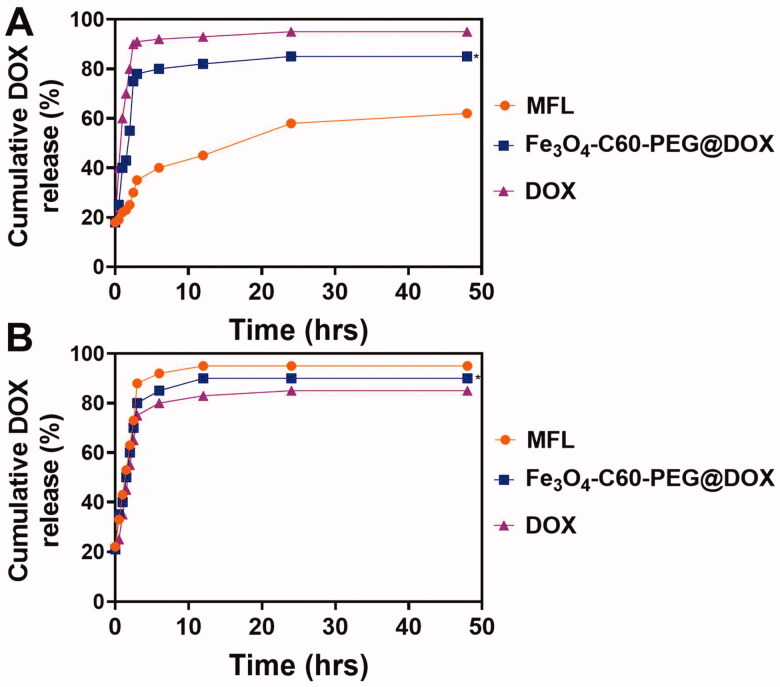
DOX release profiles from the nanocomposites at different temperatures (37 °C and 42 °C). Data are presented as means ± standard deviation (SD) (*n* = 3) (*p< .05).

We hatched the nanocomposites in sodium dodecyl sulfate solutions to assess *in vitro* DOX release (SDS, 0.5%). Incubation at 37 °C for 1 and 8 h resulted in DOX and Fe_3_O_4_-C60-PEG_2000_@DOX achieving equilibrium. By adjusting circumstances, the MFL was produced, with an encapsulation effectiveness of 91%. Using the same circumstances, the MFL was more stable, releasing ∼10 and ∼60% of DOX, respectively, after 30 min and 48 h. (Figure 5(A)). DOX outflow from the liposome was investigated at 42 °C after the liposome stability was determined at 37 °C. At 42 °C, the MFL released around ∼100% of their DOX content after 30 minutes, whereas DPPC/Fe3O4-C60-PEG2000@DOX released ∼72% in the initial five minutes of incubated and after 30 min it was reached ∼100% (Figure 5(B)). We can regulate the drugs release ratio of thermoresponsive liposomes by altering the temperatures.

### 3.2. Inhibition efficiency on HT-29 metastasis cells

**Figure 6.**
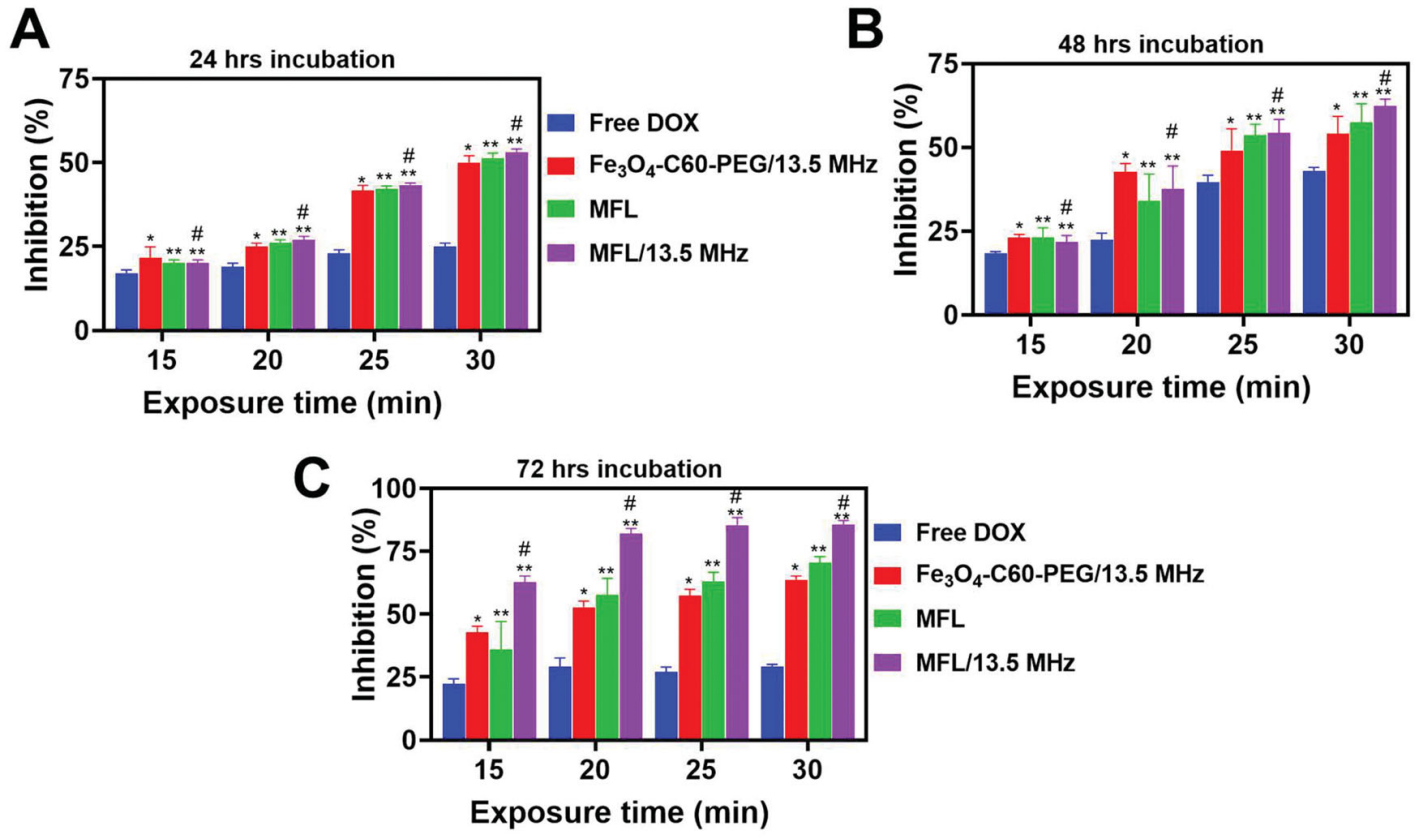
*In vitro* cytotoxicity analysis. (A–C) DOX, Fe_3_O_4_-C60-PEG_2000_/DOX and MFL on HT-29 liver metastasis cancer cells at different incubation time (24 h, 48 h, and 72 h). Data are presented as means ± standard deviation (SD) (*n* = 3) (*p< .05, **p< .05). *p< .01 vs. DOX group: ^#^*p*< .05 vs. the MFL.

According to Figure 6(A–C), MFL induced cytotoxicity in HT-29 metastasis cells. On HT-29 metastasis cells, the MFL showed greater inhibition effectiveness when irradiated with 13.56 MHz radio frequency than when not illuminated. Fe_3_O_4_-C60-PEG_2000_@DOX liposomes were discovered to be significantly different from MFLs. DSPE-PEG2000-folate may enter cells more quickly as an active targeted delivery method, and its outcomes are considerably improved than those of the other groups. HT-29 metastasis cells were inhibited more effectively by the MFL at all periods, showing that the drug delivery frameworks may carry extra drugs into cancerous cells and improve HT-29 metastasis cell inhibition.

### 3.3. Cellular uptake of DOX by HT-29 cell line

**Figure 7.**
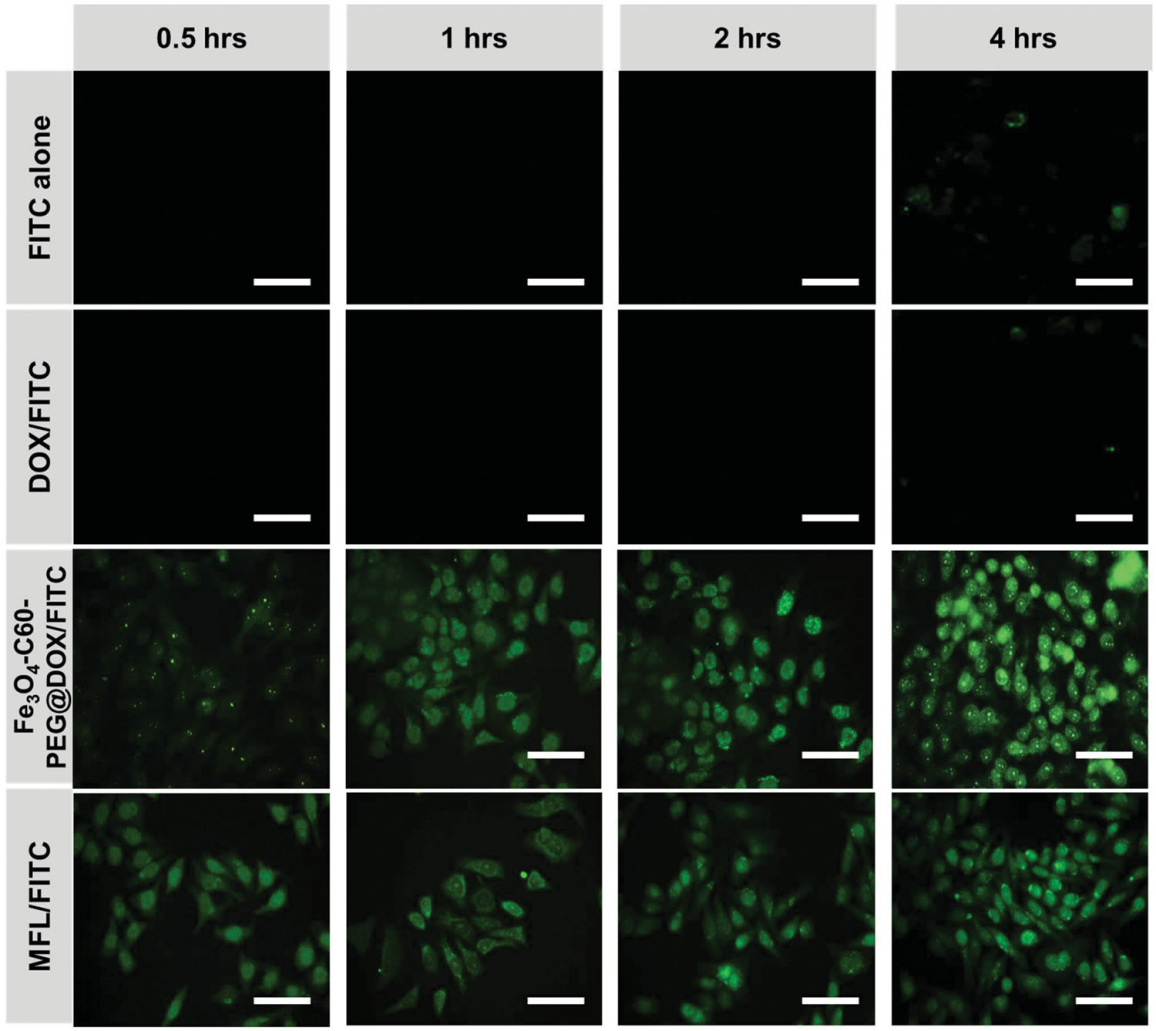
Fluorescence microscopic images of HT-29 cancer cells. FITC alone, DOX/FITC, Fe_3_O_4_-C60-PEG_2000_/DOX/FITC, MFL/FITC at different hours (0.5 h, 1 h, 2 h, and 4 h). Scale bar 100 µm. Data are presented as means of *n* = 6.

Fe_3_O_4_-C60-PEG_2000_ was tagged with FITC via – stacking of C60 to monitor its whereabouts inside cells. Cells with fluorescent-labeled C60 were tracked using fluorescence microscopy (Figure 7). In HT-29 metastasis cells treated only with FITC, very little FITC was detected, showing that FITC cannot penetrate HT-29 cells independently. The HT-29 cells could absorb the MFLs/FITC in a time-dependent fashion (Figure 7). HT-29 metastasis cells absorbed the MFLs/FITC better than Fe_3_O_4_-C60-PEG_2000_@DOX/FITC. As a result of the different susceptibilities of three DOX formulations to HT-29 metastasis cells, this variation in absorption might be explained.

### 3.4. Generation of intracellular ROS

**Figure 8.**
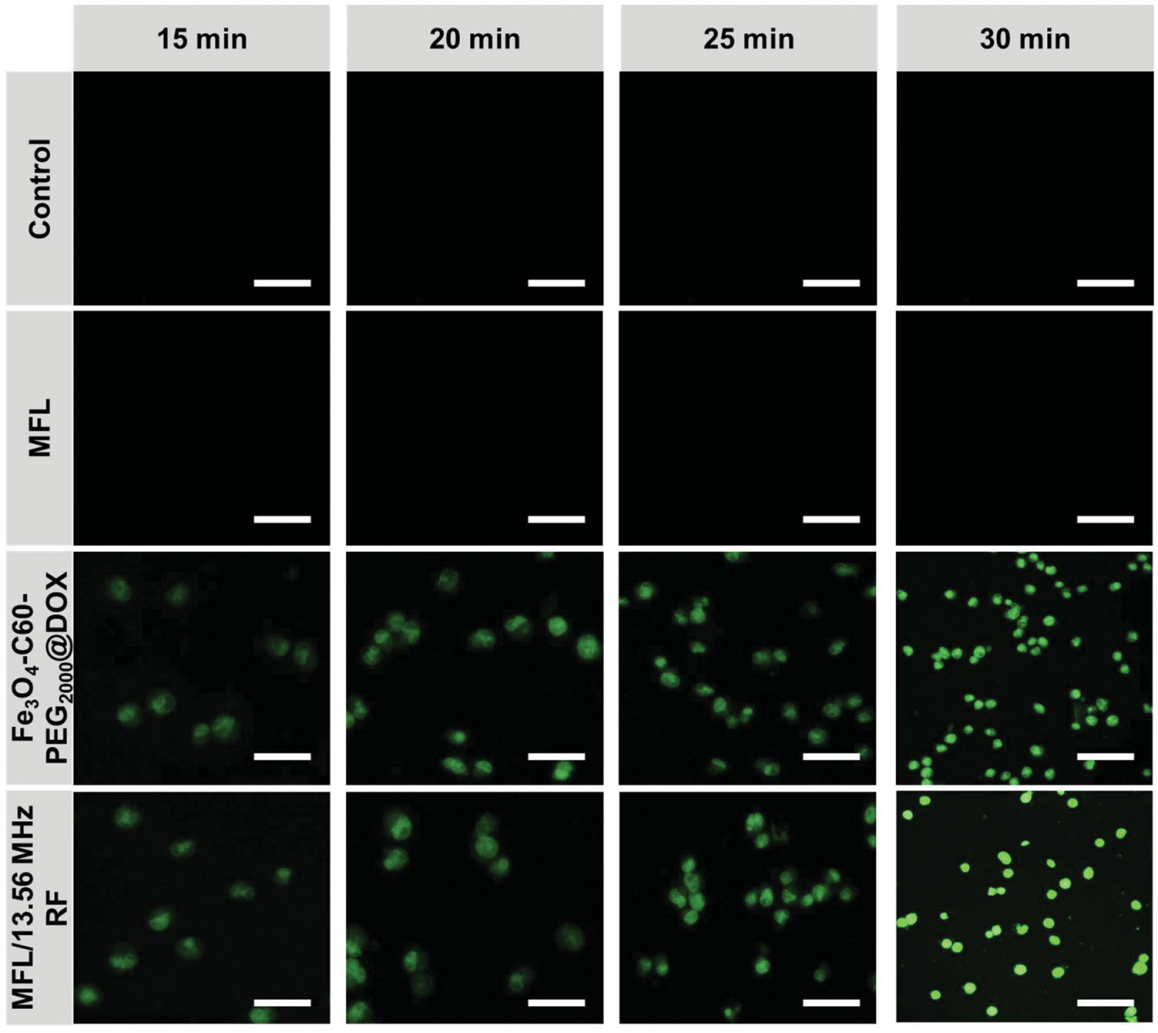
Observation of ROS generation in HT-29 cancer cells. ROS by DCFH-DA staining with Control, MFL, Fe_3_O_4_-C60-PEG_2000_/DOX/13.56 MHz RF, MFL/13.56 MHz RF. Scale bar 100 µm. Data are presented as means of *n* = 6.

An important indication of RTT is the amount of intracellular ROS. Fe_3_O_4_-C60-PEG_2000_@DOX and MFL under RF 13.56 MHz generated intracellular ROS. When HT-29 metastasis cells were treated with Fe_3_O_4_-C60-PEG_2000_@DOX and MFL, ROS generation was detected using a fluorescent DCFH-DA probe (Figure 8). Cancer cells subjected to a 300 W of 13.56 MHz RF for 15 min, 20 min, 25 min, and 30 min displayed green DCFH fluorescence, but without irradiation or untreated cells demonstrated minimal DCFH green fluorescence shown in Figure 8. HT-29 metastasis cells treatment with the MFL after radiofrequency ablation showed green DCFH fluorescence, suggesting that the MFL substantially enhanced the RTT effectiveness of DOX compared to those treated with them without RF. Fe_3_O_4_-C60-PEG_2000_@DOX and MFLs, on the other hand, did not show any significant differences after RF exposure.

### 3.5. Cell apoptosis investigation

**Figure 9.**
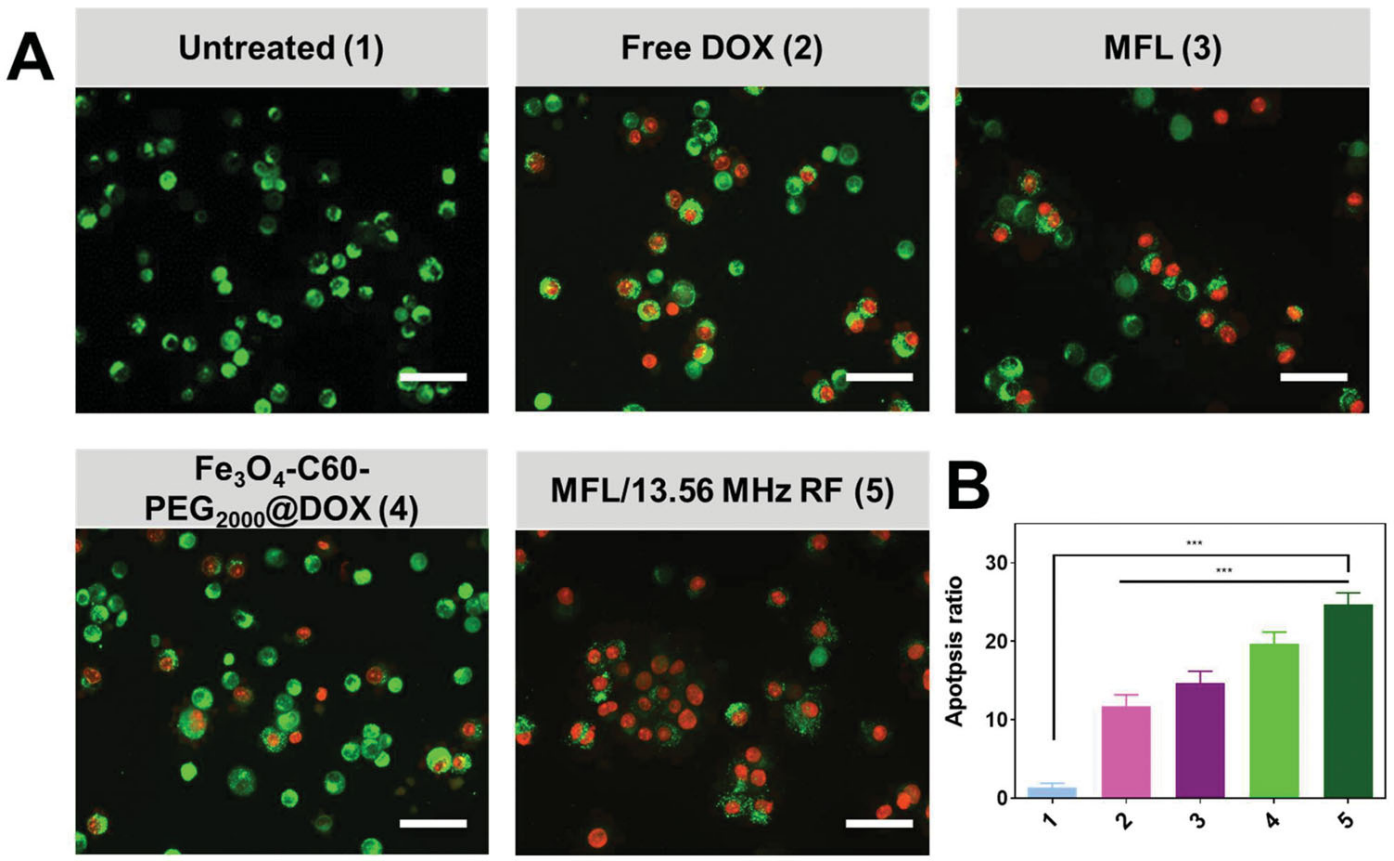
Dual AO-EB staining in HT-29 cancer cells. (A) Dual staining with untreated (1), free DOX (2), MFL (3), Fe_3_O_4_-C60-PEG_2000_/DOX/13.56 MHz RF (4), and MFL/13.56 MHz RF (5). Scale bar 100 µm. (B) Percentage of the apoptosis ratio. Data are presented as means ± standard deviation (SD) (*n* = 3); ***p< .001.

A variety of circumstances can cause apoptosis. In turn, this leads to an upstream cascade response of apoptosis, which is one of the most significant factors in promoting it. Using the AO/EB staining analysis, it is possible to determine the lysosomal membrane integrity in HT-29 metastasis cells using the AO/EB staining analysis (Kasibhatla et al., 2006; Liu et al., 2015; Zhang et al., 2019; Albayrak et al., 2021). The intact lysosomal membrane reveals green fluorescent nuclei and red fluorescent cytoplasm around the cell nucleus (Figure 9(A)). Fluorescence from a damaged lysosomal membrane is solely green. When DOX, MFL, Fe_3_O_4_-C60-PEG_2000_@DOX, and MFL/13.56 MHz RF were added to HT-29 metastasis cells, the lysosomal membrane remained intact and fluoresced red and green, displaying solely green fluorescence (Figure 9(B)).

**Figure 10.**
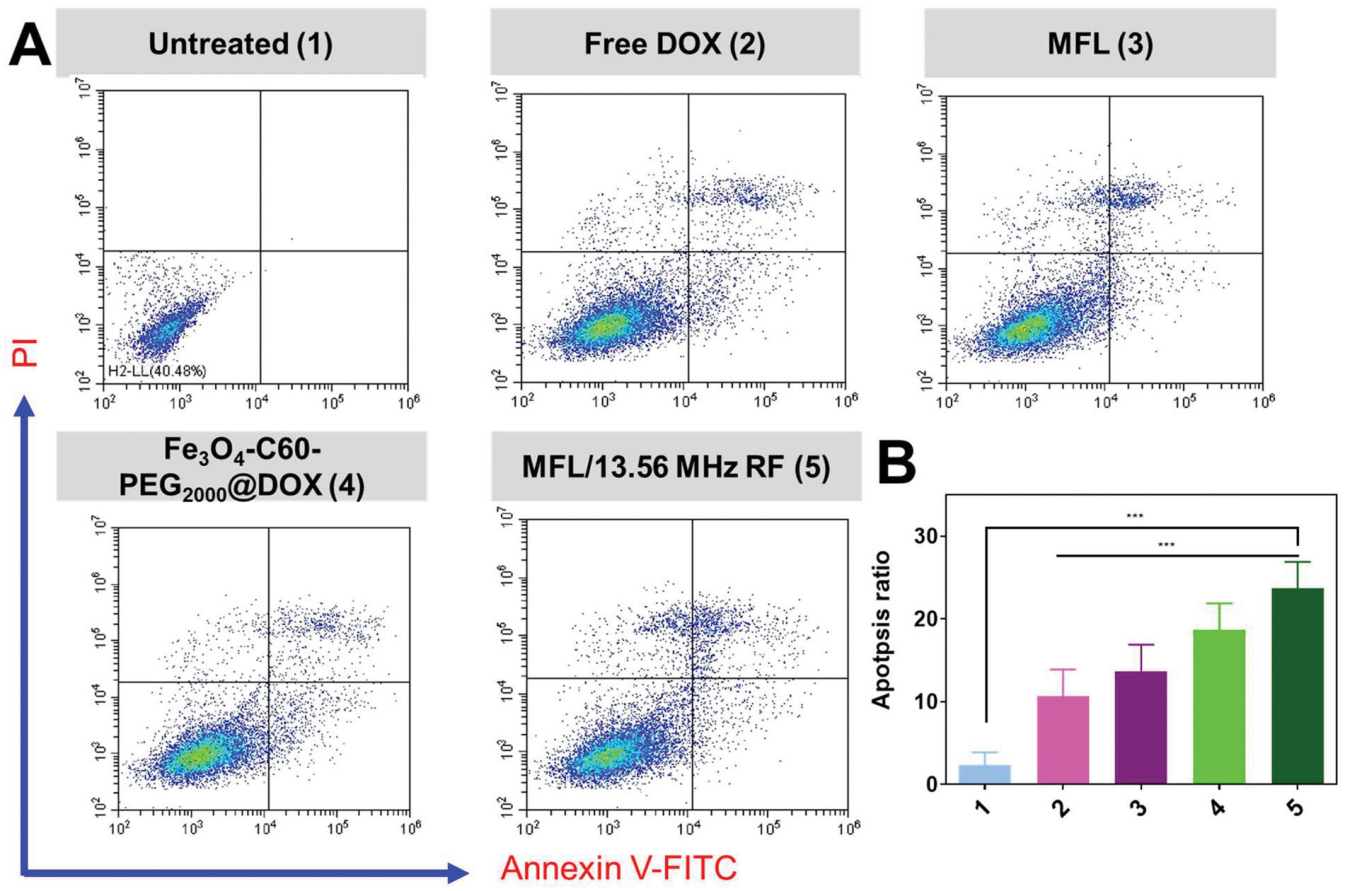
Flow cytometry Annexin FITC-V/PI staining in HT-29 cancer cells. (A) Annexin FITC-V/PI staining with untreated (1), free DOX (2), MFL (3), Fe_3_O_4_-C60-PEG_2000_/DOX/13.56 MHz RF (4), and MFL/13.56 MHz RF (5). (B) Percentage of the apoptosis ratio. Data are presented as means ± standard deviation (SD) (*n* = 3); ***p< .001.

This Annexin V-FITC/PI kit detects apoptosis in HT-29 cells by using Annexin V-FITC/PI as the apoptosis marker. Apoptosis and necrosis % ages are shown in Figure 10(A) after DOX treatment. The next treated with 1.5% of Fe_3_O_4_-C60-PEG_2000_@DOX/13.56 MHz RF and the 6.0% of MFL/13.56 MHz RF substantially enhanced the ratio of HT-29 metastasis cells in the apoptotic quadrant compared to the DOX alone (0.5%). The findings showed the cells were after a 24-h treatment with Fe_3_O_4_-C60-PEG_2000_@DOX/13.56 MHz RF, MFL/13.56 MHz RF, or the MFL in the apoptotic quadrant. When combined with RF, the MFL produced a much higher level of cell death. The *in vitro* cytotoxicity and cell death investigation of DOX formulations revealed that RF could create MFL to increase cellular internalization of drug distribution frameworks, drug release therapy representatives into the cytoplasm of the cells, and attain more significant inhibitory effects in the *in vitro* examination (Figure 10(B)).

### 3.6. *In vivo* animal model of HT-29 cells

**Figure 11.**
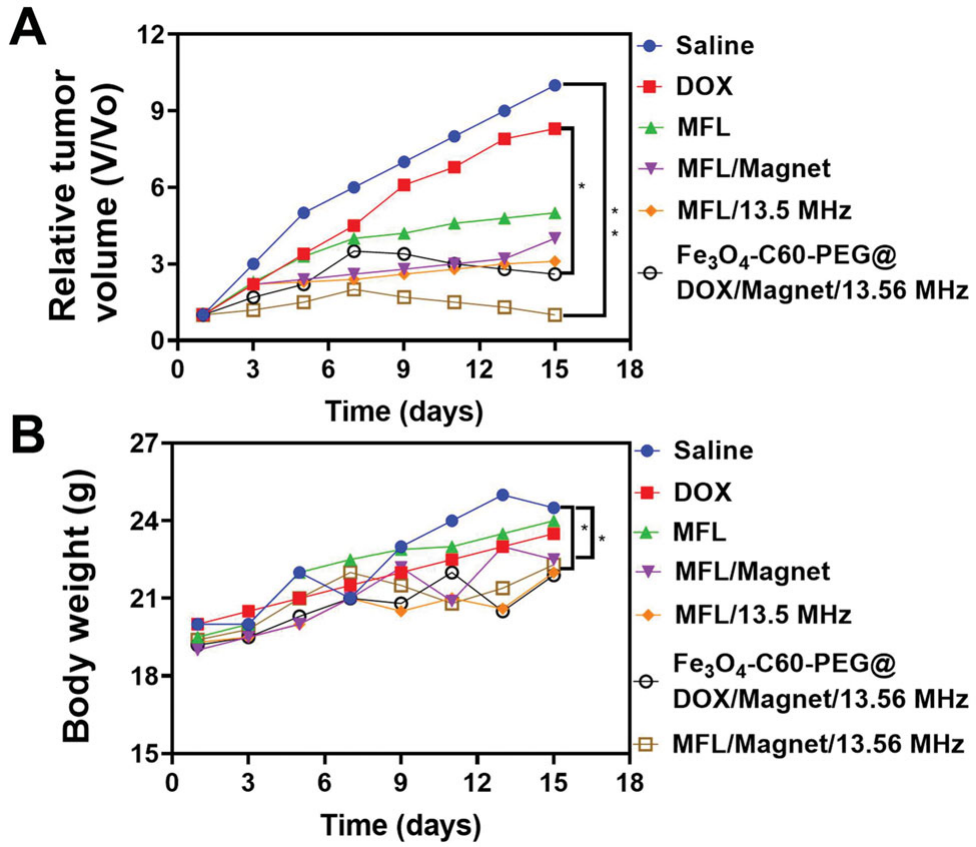
*In vivo* antitumor efficacy on HT-29 liver metastasis cancer cells. (A) Average tumor volume of the mice with various formulations within the treatment of day 14. (B) Body weight changes of the mice with various formulations within the treatment of day 14. Data are presented as means ± standard deviation (SD) (*n* = 3) (**p< .05, *p< .05).

In the experiment, the tumor temperature reached 44–46 °C using RF (20 min, 300 W). Therefore, the medication might be released from MFL, based on these findings. A comparative effectiveness analysis was undertaken to determine the *in vivo* therapeutic efficacy of the 13.56 MHz RF MFL in humans. Figure 11(A) shows the variations in tumor volume as a function of time. After 14 days of treatment with NPs, the relative tumor volume (*V*/*V*0) in the control group, while the Fe_3_O_4_-C60-PEG_2000_@DOX/magnet/13.56 MHz RF values, MFL/magnet, and MFL/13.56 MHz RF groups. According to the results, mice treated with MFL/magnet/13.56 MHz RF had much smaller tumors than mice treated with other treatment groups. RTT employed C60 to release thermoresponsive liposomes and attain *in vivo* tumor therapy effectiveness with C60. MFL magnetic targeting characteristics allowed them to reach the tumor location more efficiently than non-magnetic liposomes, resulting in a greater RTT effectiveness than without a magnetic group (Figure 11(B)). The MFL/magnet/13.56 MHz RF inhibited the development of malignant tissue with significant efficacy. This great therapeutic effectiveness is a result of the high levels of DOX and C60 in tumor cells. Even though severe toxicity generally results in weight loss, all groups of mice gained weight throughout treatment (Figure S2), indicating that the therapies' toxicity was not apparent. Tumor tissue from different treatment groups was examined histologically on day 14 administration (Figure S2) and suggested that cancer cells in the untreated (saline) group were overgrowing and had an intact morphology. Although necrotic cells, fragmentation, and lysis appeared in the MFL/magnet/13.56 MHz RF group, nearby was no way evident disparity among the rest of the groups.

### 3.7. Assessment of the pharmacokinetics

**Figure 12.**
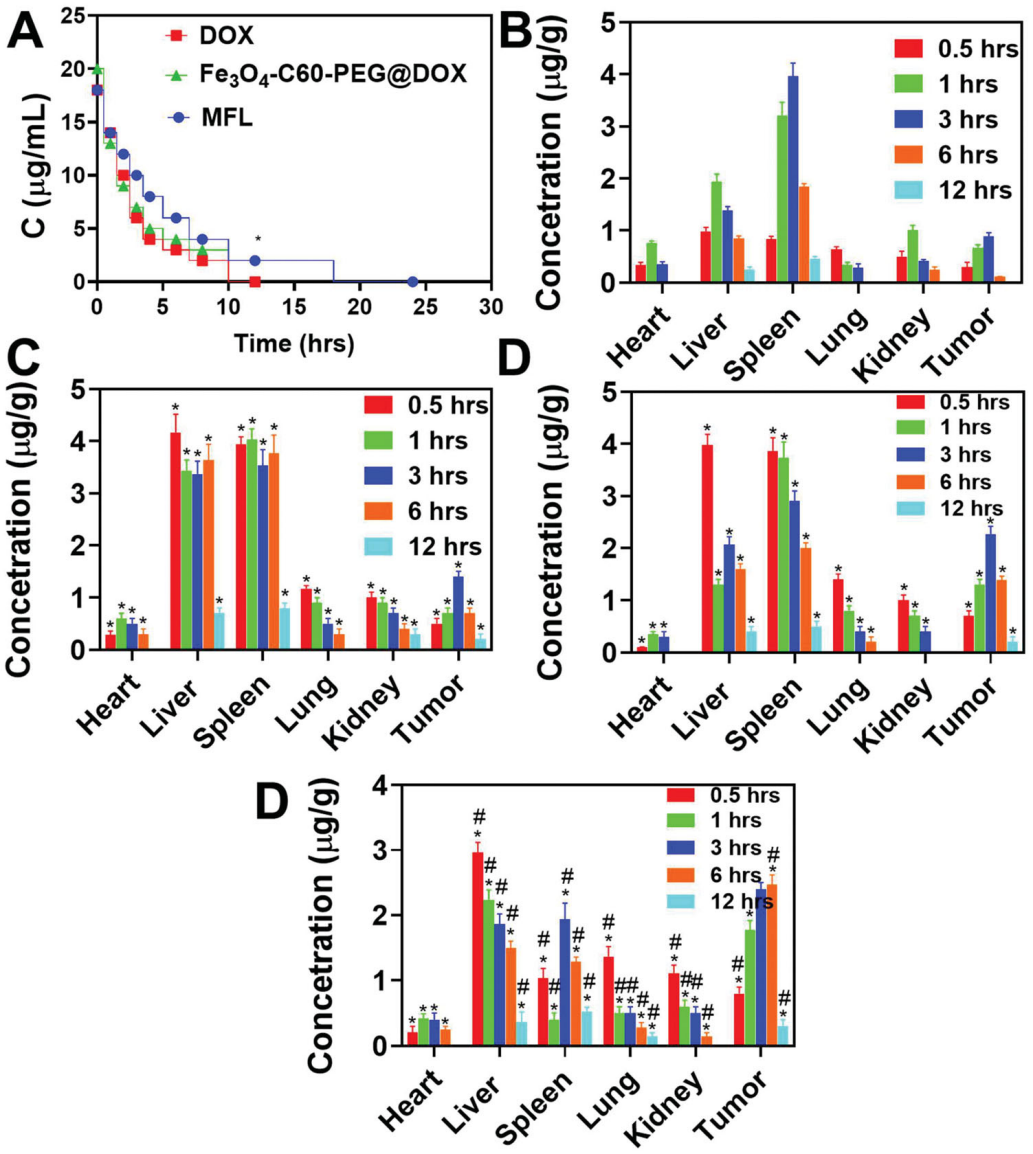
Assessment of the tissue distribution and drug time curve. (A) A mean concentration of DOX in plasma after administration (i.v.) of Fe_3_O_4_-C60-PEG_2000_/DOX and MFL. (B–D) Biodistribution of tumor-bearing mice at various hours after administration (i.v.) of DOX. MFL/DOX, Fe_3_O_4_-C60-PEG_2000_/DOX/magnet, MFL/DOX/magnet. Data are presented as means ± standard deviation (SD) (*n* = 3) (*p< .05). *p< .01 vs. DOX group: ^#^*p*<.05 vs. the MFL.

As part of the work, HPLC was used to analyze blood samples from C57 mice that had been injected with the MFL, PEG_2000_/C60-Fe_3_O_4_/DOX, or DOX at different periods. A two-compartment model was used to compute the pharmacokinetic parameters. As a result of treatment, DOX decreased more rapidly than MFL or Fe_3_O_4_-C60-PEG_2000_@DOX (Figure 12(A)). The area under the curve (AUC) of MFL (54.36 µg h/mL) was almost twofold more considerable than that of Fe_3_O_4_-C60-PEG_2000_@DOX (25.12 µg h/mL) in comparison to Fe_3_O_4_-C60-PEG_2000_@DOX. They were 19.21 L h/kg for the MFL and 41.01 L h/kg for Fe_3_O_4_-C60-PEG_2000_@DOX, respectively. MFL mean residence time (MRT) was approximately twice that of Fe_3_O_4_-C60-PEG_2000_@DOX (2.14 h), showing that MFL considerably enhanced drug blood circulation time.

### 3.8. Biodistribution

We investigated how DOX is distributed in tumors and other organs to understand the success of several DOX cancer treatments (DOX, MFL, C60-Fe_3_O_4_-PEG_2000_/DOX, and MFL/magnet). The distributions of DOX in the four formulations (Figure 12(B–D)) differed significantly. Three hours after injection, the differences in DOX distribution became most apparent. DOX absorption in tumors was significantly greater with Fe_3_O_4_-C60-PEG_2000_@DOX and MFL than with DOX and the MFL. The DOX level in tumors (3 h) was more significant in the MFL group than in the Fe_3_O_4_-C60-PEG_2000_@DOX/magnet group by around 2.0- and 1.7-folds, respectively. For the MFLs/magnets, the increased drug transport efficiency to tumors was remarkable and accountable for the increased tumor control efficiency in RTT. This is likely because folate-DSPE-PEG_2000_ as an effective, affecting drug distribution strategy and magnetic focus combined can limit the distributions of the MFL in the rest of the organs and enhance distributions in cancer to attain superior liver cancerous therapy.

## 4. Conclusions

A temperature-sensitive folate-targeted DOX-containing magnetic liposome has been effectively fabricated for thermo-chemotherapy of liver cancer. Magnetite and DOX were effectively encapsulated in liposomes with excellent efficiency. Magnetic fields promoted folate receptor-mediated absorption of the MFL into tumor cells, and they were temperature-sensitive. The MFL characteristics allowed them to destroy tumor cells more effectively than non-magnetic folate-targeted liposomes. Liver metastasis in HT-29 colon cancer metastasis cells also became more cytotoxic because of the combined use of RF, magnetic targeting, and folate active targeting. We anticipate that a persistent magnetic gradient field will increase the physical targeting of a thermo-chemotherapy formulation for targeted cancer treatment. Compared to free DOX, adverse effects should be reduced. We anticipate growing cellular absorption by tumor cells through the synergistic impact of RTT, biological targeting, and magnetic targeting.

## Supplementary Material

Supplemental Material

## References

[CIT0001] Albayrak D, Doğanlar O, Erdoğan S, et al. (2021). Naringin combined with NF-κB inhibition and endoplasmic reticulum stress induces apoptotic cell death via oxidative stress and the PERK/eIF2α/ATF4/CHOP axis in HT29 colon cancer cells. Biochem Genet 59:159–84. 32979141 10.1007/s10528-020-09996-5

[CIT0002] Balaji S, Mohamed Subarkhan MK, Ramesh R, et al. (2020). Synthesis and structure of arene Ru(II) N∧O-chelating complexes: in vitro cytotoxicity and cancer cell death mechanism. Organometallics 39:1366–75.

[CIT0003] Bale R, Putzer D, Schullian P. (2019). Local treatment of breast cancer liver metastasis. Cancers 11:1341. 10.3390/cancers11091341 PMC6770644 31514362

[CIT0004] Barani M, Bilal M, Rahdar A, et al. (2021). Nanodiagnosis and nanotreatment of colorectal cancer: an overview. J Nanoparticle Res 23:18.

[CIT0005] Beyk J, Tavakoli H. (2019). Selective radiofrequency ablation of tumor by magnetically targeting of multifunctional iron oxide-gold nanohybrid. J Cancer Res Clin Oncol 145:2199–209. 31309302 10.1007/s00432-019-02969-1 PMC11810371

[CIT0006] Chowdhury N, Chaudhry S, Hall N, et al. (2020). Targeted delivery of doxorubicin liposomes for Her-2+ breast cancer treatment. AAPS PharmSciTech 21:202. 32696338 10.1208/s12249-020-01743-8 PMC7995642

[CIT0007] Chung H-J, Lee H-K, Kwon KB, et al. (2018). Transferrin as a thermosensitizer in radiofrequency hyperthermia for cancer treatment. Sci Rep 8:13505. 30202000 10.1038/s41598-018-31232-9 PMC6131143

[CIT0008] Chung SR, Suh CH, Baek JH, et al. (2017). Safety of radiofrequency ablation of benign thyroid nodules and recurrent thyroid cancers: a systematic review and meta-analysis. Int J Hyperthermia 33:920–30. 28565997 10.1080/02656736.2017.1337936

[CIT0009] Dana P, Bunthot S, Suktham K, et al. (2020). Active targeting liposome-PLGA composite for cisplatin delivery against cervical cancer. Colloids Surf B Biointerfaces 196:111270. 32777659 10.1016/j.colsurfb.2020.111270

[CIT0010] Ding X, Yin C, Zhang W, et al. (2020). Designing aptamer-gold nanoparticle-loaded pH-sensitive liposomes encapsulate morin for treating cancer. Nanoscale Res Lett 15:68. 32232589 10.1186/s11671-020-03297-x PMC7105578

[CIT0011] Fan M, Han Y, Gao S, et al. (2020). Ultrasmall gold nanoparticles in cancer diagnosis and therapy. Theranostics 10:4944–57. 32308760 10.7150/thno.42471 PMC7163431

[CIT0012] Farzin A, Etesami SA, Quint J, et al. (2020). Magnetic nanoparticles in cancer therapy and diagnosis. Adv Healthc Mater 9:1901058. 10.1002/adhm.201901058 PMC7482193 32196144

[CIT0013] Ferreira M, Sousa J, Pais A, Vitorino C. (2020). The role of magnetic nanoparticles in cancer nanotheranostics. Materials 13:266. 10.3390/ma13020266 PMC7014348 31936128

[CIT0014] Gu Z, Da Silva CG, Van der Maaden K, et al. (2020). Liposome-based drug delivery systems in cancer immunotherapy. Pharmaceutics 12:1054. 10.3390/pharmaceutics12111054 PMC7694212 33158166

[CIT0015] Gulzar A, Gai S, Yang P, et al. (2015). Stimuli responsive drug delivery application of polymer and silica in biomedicine. J Mater Chem B 3:8599–622. 32262717 10.1039/c5tb00757g

[CIT0016] Han H, Eigentler TW, Wang S, et al. (2020). Design, implementation, evaluation and application of a 32-channel radio frequency signal generator for thermal magnetic resonance based anti-cancer treatment. Cancers 12:1720. 10.3390/cancers12071720 PMC7408155 32605322

[CIT0017] Jose A, Surendran M, Fazal S, et al. (2018). Multifunctional fluorescent iron quantum clusters for non-invasive radiofrequency ablation of cancer cells. Colloids Surf B Biointerfaces 165:371–80. 29525697 10.1016/j.colsurfb.2018.02.058

[CIT0018] Kasibhatla S, Amarante-Mendes GP, Finucane D, et al. (2006). Acridine orange/ethidium bromide (AO/EB) staining to detect apoptosis. Cold Spring Harb Protoc 2006:pdb.prot4493.

[CIT0019] Khalifehzadeh R, Arami H. (2020). Biodegradable calcium phosphate nanoparticles for cancer therapy. Adv Colloid Interface Sci 279:102157. 32330734 10.1016/j.cis.2020.102157 PMC7261203

[CIT0020] Kim B, Shin J, Wu J, et al. (2020). Engineering peptide-targeted liposomal nanoparticles optimized for improved selectivity for HER2-positive breast cancer cells to achieve enhanced in vivo efficacy. J Control Release 322:530–41. 32276005 10.1016/j.jconrel.2020.04.010 PMC7932755

[CIT0021] Ledezma-Gallegos F, Jurado R, Mir R, et al. (2020). Liposomes co-encapsulating cisplatin/mifepristone improve the effect on cervical cancer: in vitro and in vivo assessment. Pharmaceutics 12:897. 10.3390/pharmaceutics12090897 PMC7558205 32971785

[CIT0022] Liu K, Liu P, Liu R, Wu X. (2015). Dual AO/EB staining to detect apoptosis in osteosarcoma cells compared with flow cytometry. Med Sci Monit Basic Res 21:15–20. 25664686 10.12659/MSMBR.893327 PMC4332266

[CIT0023] Löffler MW, Nussbaum B, Jäger G, et al. (2019). A non-interventional clinical trial assessing immune responses after radiofrequency ablation of liver metastases from colorectal cancer. Front Immunol 10:2526. 31803175 10.3389/fimmu.2019.02526 PMC6877671

[CIT0024] Lv Q, Cheng L, Lu Y, et al. (2020). Thermosensitive exosome-liposome hybrid nanoparticle-mediated chemoimmunotherapy for improved treatment of metastatic peritoneal cancer. Adv Sci 7:2000515. 10.1002/advs.202000515 PMC7509655 32999828

[CIT0025] Mansoori B, Mohammadi A, Abedi-Gaballu F, et al. (2020). Hyaluronic acid-decorated liposomal nanoparticles for targeted delivery of 5-fluorouracil into HT-29 colorectal cancer cells. J Cell Physiol 235:6817–30. 31989649 10.1002/jcp.29576 PMC7384933

[CIT0026] Marques AC, Costa PJ, Velho S, Amaral MH. (2020). Functionalizing nanoparticles with cancer-targeting antibodies: a comparison of strategies. J Control Release 320:180–200. 31978444 10.1016/j.jconrel.2020.01.035

[CIT0027] Mauri G, Gennaro N, Lee MK, Baek JH. (2019). Laser and radiofrequency ablations for benign and malignant thyroid tumors. Int J Hyperthermia 36:13–20. 31537159 10.1080/02656736.2019.1622795

[CIT0028] Mignani S, Shi X, Guidolin K, et al. (2021). Clinical diagonal translation of nanoparticles: case studies in dendrimer nanomedicine. J Control Release 337:356–70. 34311026 10.1016/j.jconrel.2021.07.036

[CIT0029] Mohamed Kasim MS, Sundar S, Rengan R. (2018). Synthesis and structure of new binuclear ruthenium(II) arene benzil bis(benzoylhydrazone) complexes: investigation on antiproliferative activity and apoptosis induction. Inorg Chem Front 5:585–96.

[CIT0030] Mohamed Subarkhan MK, Ramesh R, Liu Y. (2016). Synthesis and molecular structure of arene ruthenium(II) benzhydrazone complexes: impact of substitution at the chelating ligand and arene moiety on antiproliferative activity. New J Chem 40:9813–23.

[CIT0031] Mohamed Subarkhan MK, Ren L, Xie B, et al. (2019). Novel tetranuclear ruthenium(II) arene complexes showing potent cytotoxic and antimetastatic activity as well as low toxicity in vivo. Eur J Med Chem 179:246–56. 31255925 10.1016/j.ejmech.2019.06.061

[CIT0032] Naumenko VA, Vodopyanov SS, Vlasova KY, et al. (2021). Intravital imaging of liposome behavior upon repeated administration: a step towards the development of liposomal companion diagnostic for cancer nanotherapy. J Control Release 330:244–56. 33333122 10.1016/j.jconrel.2020.12.014

[CIT0033] Oberacker E, Kuehne A, Oezerdem C, et al. (2020). Radiofrequency applicator concepts for thermal magnetic resonance of brain tumors at 297 MHz (7.0 Tesla). Int J Hyperthermia 37:549–63. 32484019 10.1080/02656736.2020.1761462 PMC8352381

[CIT0034] Palussière J, Chomy F, Savina M, et al. (2018). Radiofrequency ablation of stage IA non-small cell lung cancer in patients ineligible for surgery: results of a prospective multicenter phase II trial. J Cardiothorac Surg 13:91. 30143031 10.1186/s13019-018-0773-y PMC6109264

[CIT0035] Pantano P, Harrison CD, Poulose J, et al. (2017). Factors affecting the 13.56-MHz radio-frequency-mediated heating of gold nanoparticles. Appl Spectrosc Rev 52:821–36.

[CIT0036] Patel G, Thakur NS, Kushwah V, et al. (2020). Liposomal delivery of mycophenolic acid with quercetin for improved breast cancer therapy in SD rats. Front Bioeng Biotechnol 8:631. 32612988 10.3389/fbioe.2020.00631 PMC7308462

[CIT0037] Paulides MM, Dobsicek Trefna H, Curto S, Rodrigues DB. (2020). Recent technological advancements in radiofrequency- and microwave-mediated hyperthermia for enhancing drug delivery. Adv Drug Deliv Rev 163–164:3–18. 10.1016/j.addr.2020.03.004 32229271

[CIT0038] Prasad B, Kim S, Cho W, et al. (2018). Effect of tumor properties on energy absorption, temperature mapping, and thermal dose in 13.56-MHz radiofrequency hyperthermia. J Therm Biol 74:281–9. 29801639 10.1016/j.jtherbio.2018.04.007

[CIT0039] Rangamuwa K, Leong T, Weeden C, et al. (2021). Thermal ablation in non-small cell lung cancer: a review of treatment modalities and the evidence for combination with immune checkpoint inhibitors. Transl Lung Cancer Res 10:2842–57. 34295682 10.21037/tlcr-20-1075 PMC8264311

[CIT0040] Sarangi SC, Pattnaik SS, Katyal J, et al. (2020). An interaction study of *Ocimum sanctum* L. and levetiracetam in pentylenetetrazole kindling model of epilepsy. J Ethnopharmacol 249:112389. 31739106 10.1016/j.jep.2019.112389

[CIT0041] Sathiya Kamatchi T, Mohamed Subarkhan MK, Ramesh R, et al. (2020). Investigation into antiproliferative activity and apoptosis mechanism of new arene Ru(II) carbazole-based hydrazone complexes. Dalton Trans 49:11385–95. 32776042 10.1039/d0dt01476a

[CIT0042] Shoji H, Motegi M, Takakusagi Y, et al. (2017). Chemoradiotherapy and concurrent radiofrequency thermal therapy to treat primary rectal cancer and prediction of treatment responses. Oncol Rep 37:695–704. 27959450 10.3892/or.2016.5300 PMC5355662

[CIT0043] Song S, Guo H, Jiang Z, et al. (2015). Self-assembled microbubbles as contrast agents for ultrasound/magnetic resonance dual-modality imaging. Acta Biomater 24:266–78. 26112374 10.1016/j.actbio.2015.06.025

[CIT0044] Sonju JJ, Dahal A, Singh SS, Jois SD. (2021). Peptide-functionalized liposomes as therapeutic and diagnostic tools for cancer treatment. J Control Release 329:624–44. 33010333 10.1016/j.jconrel.2020.09.055 PMC8082750

[CIT0045] Subarkhan MKM, Ramesh R. (2016). Ruthenium(II) arene complexes containing benzhydrazone ligands: synthesis, structure and antiproliferative activity. Inorg Chem Front 3:1245–55.

[CIT0046] Swami Vetha BS, Oh P-S, Kim SH, Jeong H-J. (2020). Curcuminoids encapsulated liposome nanoparticles as a blue light emitting diode induced photodynamic therapeutic system for cancer treatment. J Photochem Photobiol B 205:111840. 32146273 10.1016/j.jphotobiol.2020.111840

[CIT0047] Unnam S, Panduragaiah VM, Sidramappa MA, Muddana Eswara BR. (2019). Gemcitabine-loaded folic acid tagged liposomes: improved pharmacokinetic and biodistribution profile. Curr Drug Deliv 16:111–22. 30360740 10.2174/1567201815666181024112252

[CIT0048] Vu MN, Kelly HG, Wheatley AK, et al. (2020). Cellular interactions of liposomes and PISA nanoparticles during human blood flow in a microvascular network. Small 16:2002861. 10.1002/smll.202002861 PMC7361276 32583981

[CIT0049] Wang X-X, Li Y-B, Yao H-J, et al. (2011). The use of mitochondrial targeting resveratrol liposomes modified with a dequalinium polyethylene glycol–distearoylphosphatidyl ethanolamine conjugate to induce apoptosis in resistant lung cancer cells. Biomaterials 32:5673–87. 21550109 10.1016/j.biomaterials.2011.04.029

[CIT0050] Wust P, Kortüm B, Strauss U, et al. (2020). Non-thermal effects of radiofrequency electromagnetic fields. Sci Rep 10:13488. 32778682 10.1038/s41598-020-69561-3 PMC7417565

[CIT0051] Xiao Z, Zhuang B, Zhang G, et al. (2021). Pulmonary delivery of cationic liposomal hydroxycamptothecin and 5-aminolevulinic acid for chemo-sonodynamic therapy of metastatic lung cancer. Int J Pharm 601:120572. 33831485 10.1016/j.ijpharm.2021.120572

[CIT0052] Yang M, Li J, Gu P, Fan X. (2021). The application of nanoparticles in cancer immunotherapy: targeting tumor microenvironment. Bioact Mater 6:1973–87. 33426371 10.1016/j.bioactmat.2020.12.010 PMC7773537

[CIT0053] Yang Z, Shi J, Xie J, et al. (2020). Large-scale generation of functional mRNA-encapsulating exosomes via cellular nanoporation. Nat Biomed Eng 4:69–83. 31844155 10.1038/s41551-019-0485-1 PMC7080209

[CIT0054] Zhang N, Chen H, Liu A-Y, et al. (2016). Gold conjugate-based liposomes with hybrid cluster bomb structure for liver cancer therapy. Biomaterials 74:280–91. 26461120 10.1016/j.biomaterials.2015.10.004

[CIT0055] Zhang R, Song X, Liang C, et al. (2017). Catalase-loaded cisplatin-prodrug-constructed liposomes to overcome tumor hypoxia for enhanced chemo-radiotherapy of cancer. Biomaterials 138:13–21. 28550753 10.1016/j.biomaterials.2017.05.025

[CIT0056] Zhang W-Y, Wang Y-J, Du F, et al. (2019). Evaluation of anticancer effect in vitro and in vivo of iridium(III) complexes on gastric carcinoma SGC-7901 cells. Eur J Med Chem 178:401–16. 31202128 10.1016/j.ejmech.2019.06.003

[CIT0057] Zhou M, Chen Y, Adachi M, et al. (2015). Single agent nanoparticle for radiotherapy and radio-photothermal therapy in anaplastic thyroid cancer. Biomaterials 57:41–9. 25913249 10.1016/j.biomaterials.2015.04.013 PMC4426239

