# Supplementary material for: Synergic fabrication of multifunctional liposomes nanocomposites for improved radiofrequency ablation combination for liver metastasis cancer therapy
Source: Drug Deliv. 2022 Feb 11;29(1):506–18. doi: 10.1080/10717544.2021.2008056 (PMC8845112; doi:10.1080/10717544.2021.2008056)
Supplement: Supplemental Material [file IDRD_A_2008056_SM7831.docx]

Synergic Fabrication of Multifunctional Liposomes Nanocomposites for Improved Radiofrequency Ablation Combination for Liver Metastasis Cancer Therapy

Ning Zhang^1,#^, Yibin Wu^1,#^, Weiqi Xu^1^, Zhenjian Li^2^, Lu Wang^1,*^

^1^Department of Hepatic Surgery, Fudan University Shanghai Cancer Center, Shanghai-200032, China.

﻿^2^3D Biomedicine Science & Technology Co., Limited, Shanghai-201112, China.

﻿Ning Zhang and Yibin Wu contributed equally to this study.

Correspondence to:

Dr Lu Wang, Department of Hepatic Surgery, Fudan University Shanghai Cancer Center, Dong-An Road 270 Shanghai-200032, China.


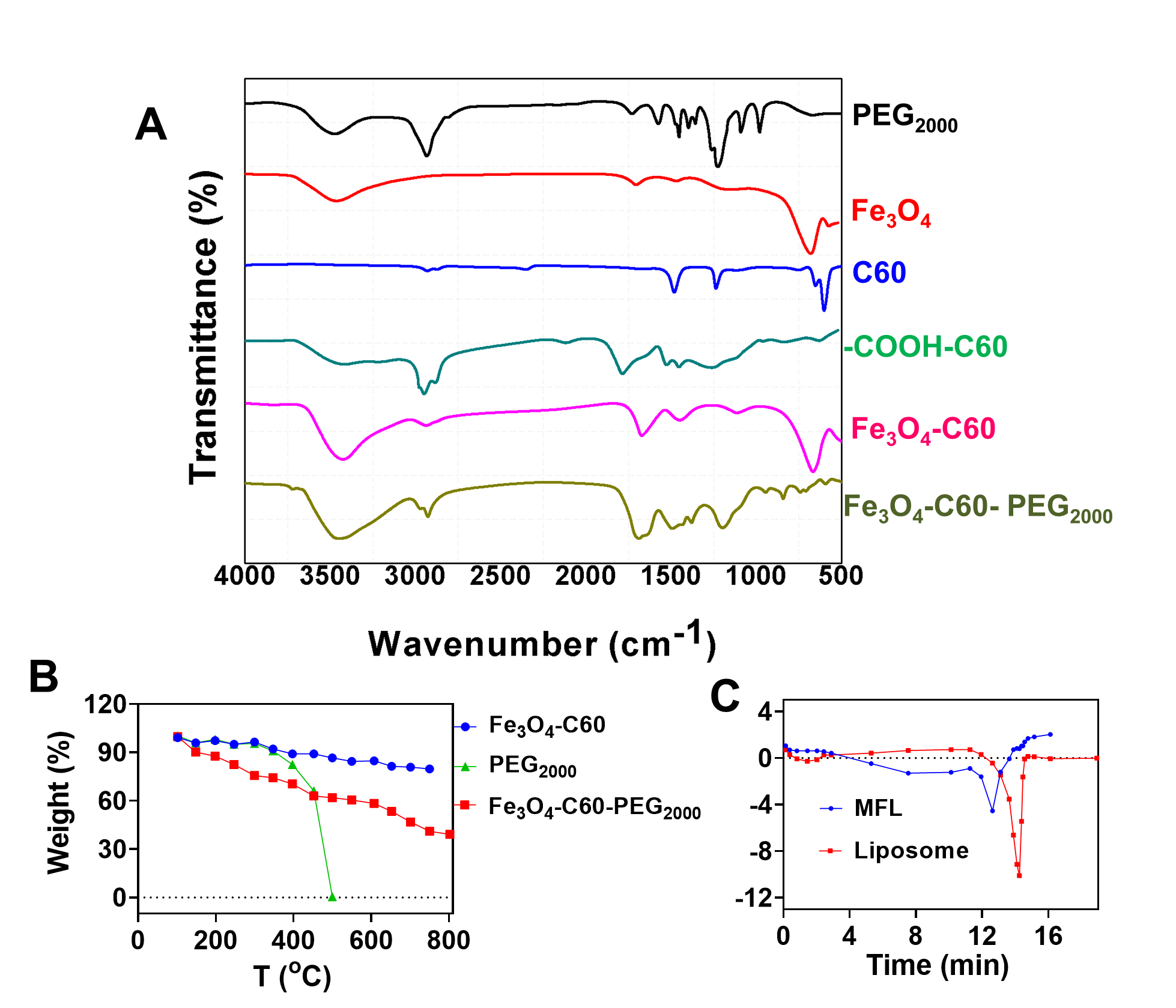


Figure S1. Nanocomposite's characterizations. A) FTIR spectra analysis of PEG_2000_, Fe_3_O_4_, C60, -COOH-C60, Fe_3_O_4_-C60, Fe_3_O_4_-C60-PEG_2000_. B) TGA analysis of Fe_3_O_4_-C60, PEG_2000_, Fe_3_O_4_-C60, Fe_3_O_4_-C60-PEG_2000_. C) DSC analysis of MFL and free liposomes.


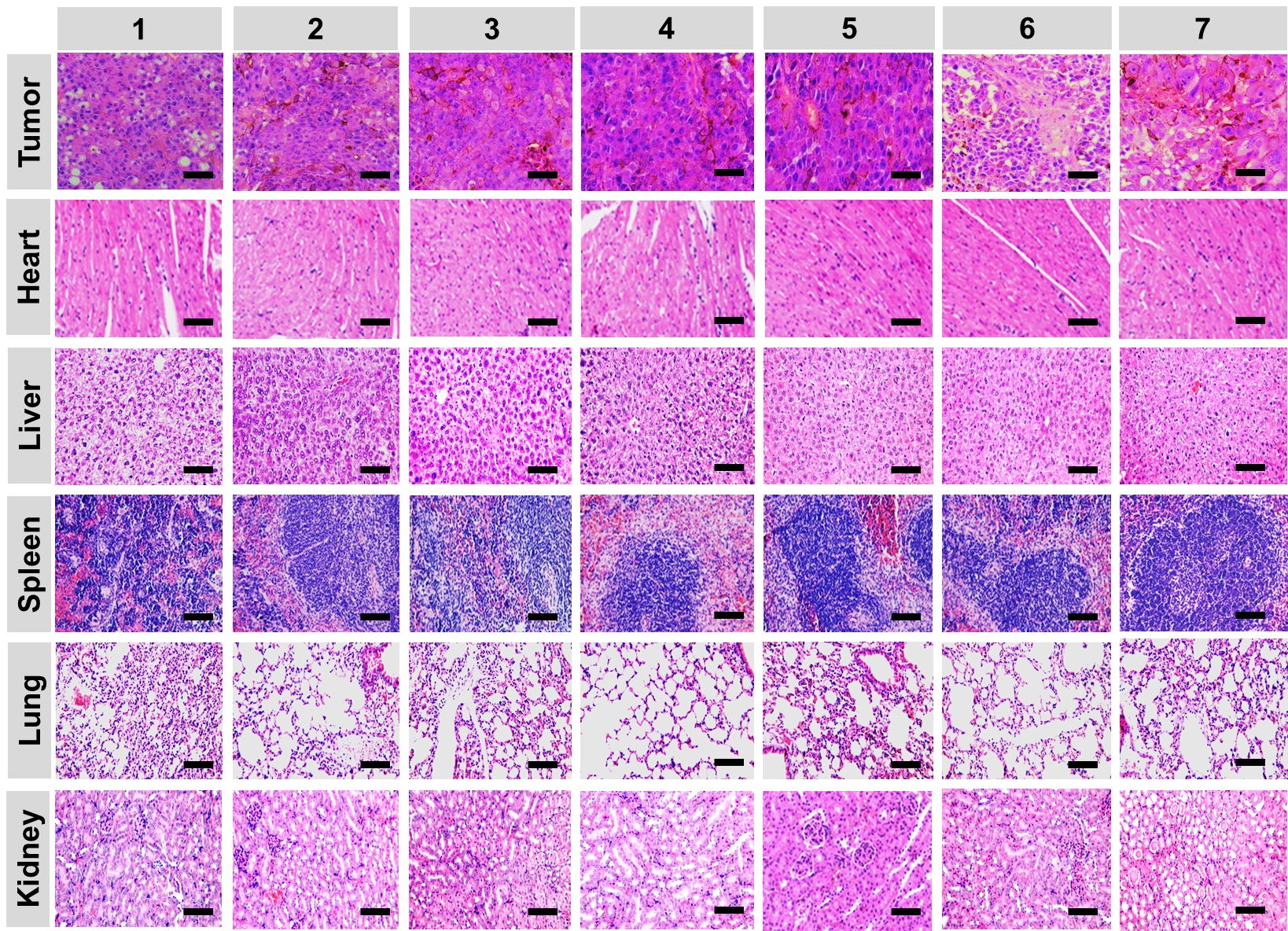


Figure S2. H&E staining of tumour tissues collected from the mice with various formulations within the treatment of day 14 on saline (**1**), DOX (**2**), MFL (**3**), MFL/13.56 MHz RF (**4**), MFL/magnet (**5**), Fe_3_O_4_-C60-PEG_2000_/DOX/magnet/13.56 MHz RF (**6**), MFL/magnet/13.56 MHz RF (**7**). Scale bar 100 µm.
